# Closure of a Complex Lower Extremity Wound With the Use of Multiple Negative Pressure Therapy Modalities

**DOI:** 10.7759/cureus.9247

**Published:** 2020-07-17

**Authors:** Elizabeth Eldenburg, Maci Pfaffenberger, Allen Gabriel

**Affiliations:** 1 Plastic Surgery, Allen Gabriel MD, Vancouver, USA

**Keywords:** plastic and reconstructive surgery, skin graft, wound, closed incisional negative pressure wound therapy, wound infections, abscess, motor vehicle collison, morel-lavallée lesions, incisional negative pressure wound therapy, morel-lavallée

## Abstract

Complex lower extremity wounds can present challenges in healing due to the cause of injury or previous surgery, presence of infection or tissue necrosis, patient comorbidities, or a combination of these factors. Negative pressure wound therapy (NPWT) modalities play a major role in the perioperative management of patients with complex wounds and their adjunctive use continues to evolve with time. In this case study, we discuss the use of adjunctive NPWT with instillation and dwell time (NPWTi-d) and closed incision negative pressure therapy (ciNPT) to assist with the management of a complex lower extremity wound. The patient was a 25-year-old female who presented with an actively draining Morel-Lavallée lesion of the left lateral thigh that she had previously sustained after being struck by a motor vehicle as a pedestrian. She was initially evaluated and admitted for the avulsion injury approximately two weeks prior to this and had a drain placed at that time. However, due to issues with compliance, she had not been re-evaluated. She now presented with a suspected infection of her left lower extremity, and was thus admitted, placed on intravenous cefazolin and underwent several rounds of excisional debridement and irrigation. The patient was then managed operatively by the plastic surgery service. This care included three rounds of tissue advancement, followed by a seven-day course of NPWTi-d. Cycles consisted of normal saline instillation with a one-second dwell time, followed by six hours of continuous negative pressure at −125 mm Hg. The patient was then taken back for a final round of reconstruction with tissue advancement. A split-thickness skin graft was used at that time to cover the remaining area of the wound that the advancement could not close. A seven-day course of ciNPT (PREVENA RESTOR BELLA•FORM™ System; 3M + KCI, San Antonio, TX) was then applied to manage the incisions and bolster the graft. This was followed by simple dressing changes several times weekly for four weeks. In this case, we demonstrate how the adjunctive use of multiple NPWT modalities resulted in a completely healed wound within two months, without any major complications.

## Introduction

Traumatic wounds are often difficult to resolve due to the risk or presence of complications, including infection, soft tissue loss, ischemia, or necrosis. The primary goal in managing traumatic wounds is to control and stabilize the acute injury, followed by decontamination and preparation of the wound bed for coverage, if this is needed. In situations such as these, we attempt to “replace like tissue with like”, and achieve anatomical reconstruction. This includes replacing the missing soft tissue layers such as the epidermis, dermis, and subcutaneous tissue. The best way to achieve this is with a distant or local flap, when available. These methods of closure are essential to restoring vascularity, providing physical protection from infection, and thus creating an optimal environment for wound healing [1]. Negative pressure wound therapy (NPWT) has not only been proven to promote tissue perfusion [2], but is also known to assist with lower extremity flap perfusion [3]. We now discuss a case in which NPWT with instillation and dwell time (NPWTi-d) and closed incision negative pressure therapy (ciNPT) were used in concert to manage and support closure of a complex left lateral thigh wound due to extensive trauma and degloving injury from a motor vehicle accident. This wound was particularly challenging, as it initially presented with infection, necrosis, extensive degloving, and soft tissue loss.

## Case presentation

A 25-year-old woman with a history of smoking and obesity presented to the ED with a fever and an infected lower extremity wound with active, purulent drainage. About two weeks prior to this, the patient sustained a degloving injury to her left lateral thigh after being struck by a motor vehicle as a pedestrian. She was thus admitted as an inpatient at which point a drain was placed due to the presence of a Morel-Lavallée lesion. On discharge from the hospital, she was instructed to follow up, however had yet to be seen.

On this most recent visit to the ED, the patient was re-admitted to the hospital for suspected infection and intravenous antibiotics were initiated with cefazolin. Cultures of the abscess revealed a necrotizing infection with methicillin-sensitive *Staphylococcus aureus* (MSSA). She underwent multiple excisional debridement and irrigation procedures as an inpatient, where her wound was noted to have extensive undermining and tissue necrosis (Video 1). As the infection continued to resolve, plastic surgery was consulted to manage the wound. To successfully close the large dead space, multiple modalities were considered, including possible wound vacuum-assisted closure (VAC) application, tissue advancement, delayed primary closure, and skin graft application.

**Video 1:** Illustration of extensive undermining This video illustrates just how extensive the degloving injury appeared on initial evaluation in the operating room just prior to placing irrigating negative pressure therapy to the left lower extremity wound.

**Figure 1:**
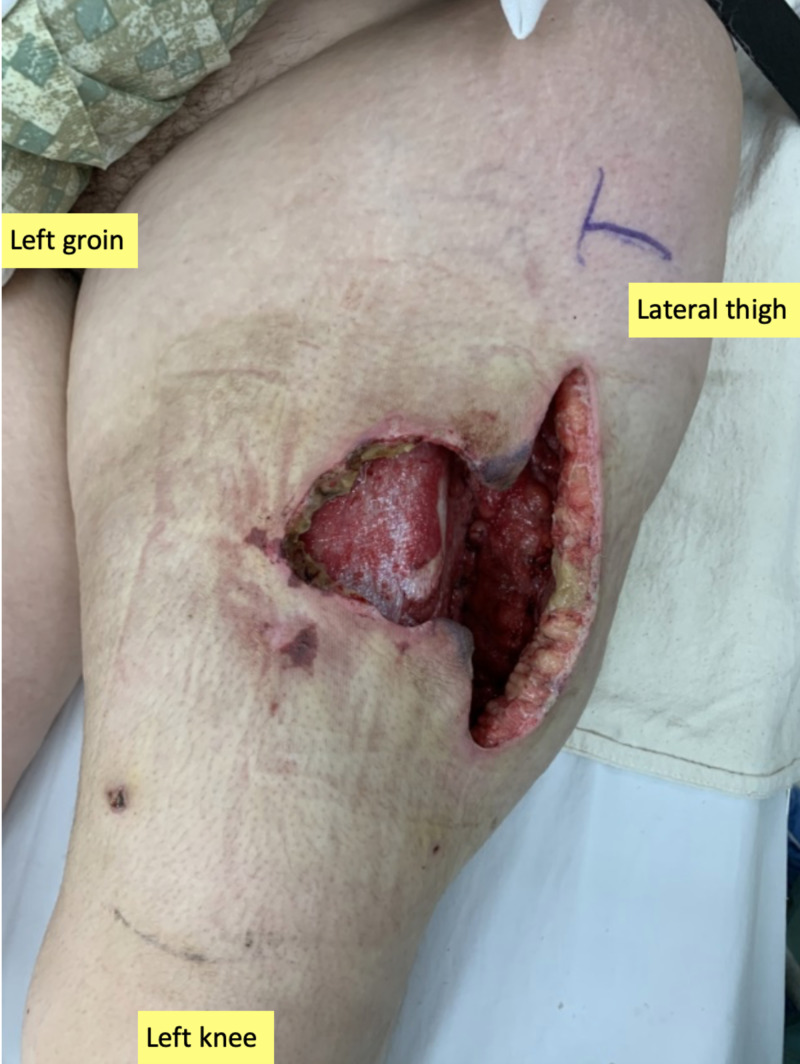
Initial presentation of the infected medial left leg wound Note the extent of the Morel-Lavallée lesion and dusky appearance of wound edges.

The plastic surgery service performed serial debridements and tissue advancement procedures, each seven days apart to collapse the large degloving injury (Figure 1).

**Figure 2:**
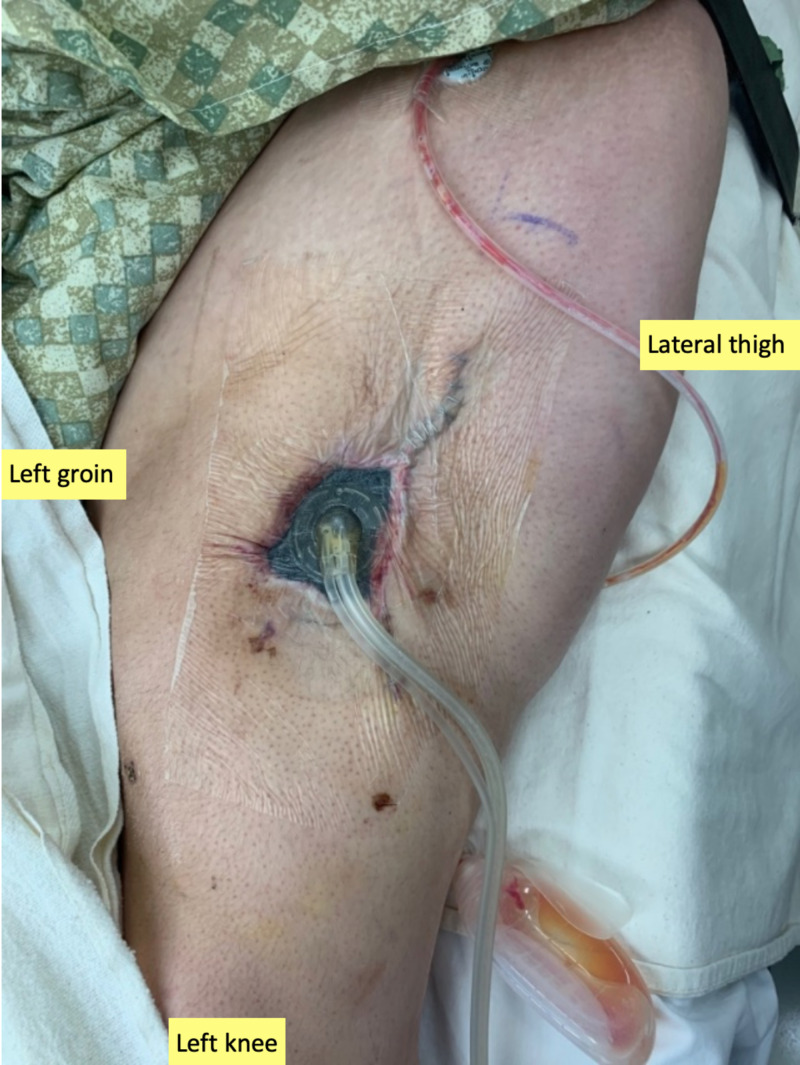
Application of irrigating negative pressure therapy Placement of negative pressure wound therapy with instillation and dwell time following tissue advancement to enhance granulation tissue formation and flap viability.

During each reconstructive surgery, tissue was rotated and advanced to obliterate the underlying dead space. Following each surgery, wound cleansing was initiated using NPWTi-d (V.A.C. VeraFlo™ Therapy; 3M + KCI, San Antonio, TX) and a reticulated open-cell foam dressing (ROCF-V; V.A.C. VeraFlo™ Dressing; 3M + KCI). Normal saline was instilled until the foam was saturated for a one-second dwell time, followed by six hours of continuous negative pressure at −125 mm Hg (Figure 2). The one-second dwell time was deliberately chosen to simulate frequent irrigation of the wound, rather than soaking it for longer periods of time.

**Figure 3:**
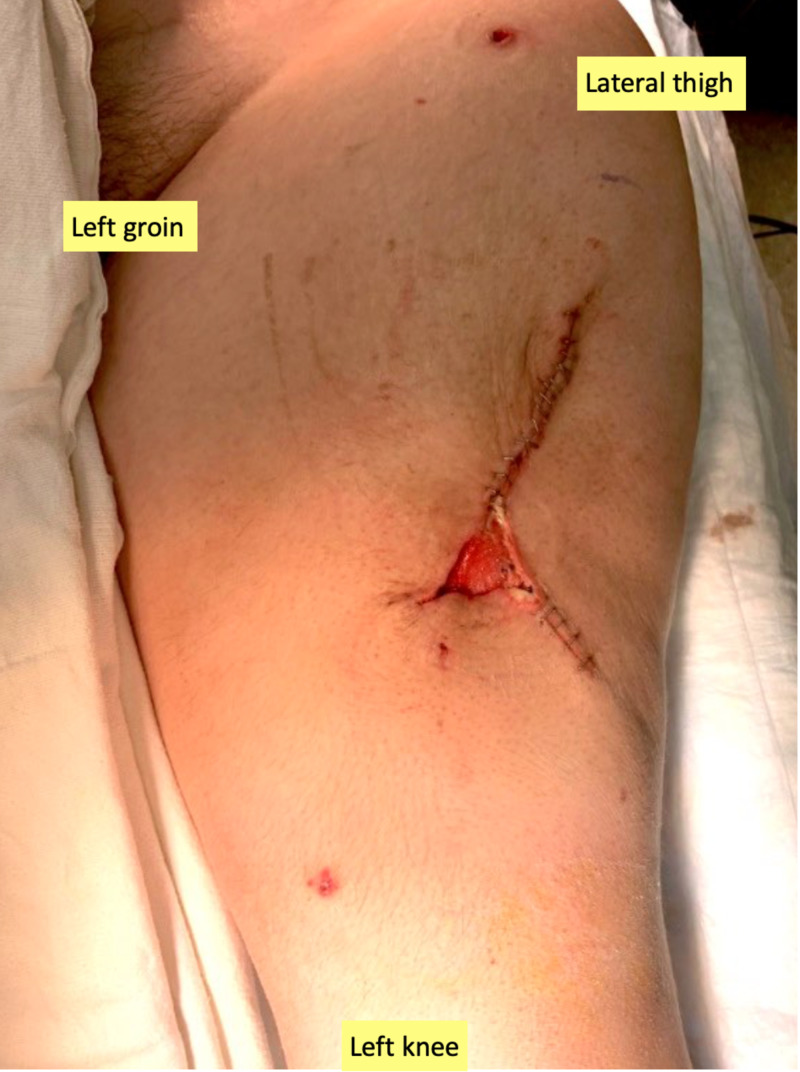
Pre-closure of the donor site with a STSG STSG, split-thickness skin graft. Appearance of the exposed wound bed during the final reconstructive surgery, just prior to closure with a STSG.

The patient was then taken back to the operating room for a final round of reconstruction with the intent to completely close the wound with application of a split-thickness skin graft (STSG). Excisional debridement was performed, and a small area of tissue was rotated to mostly collapse the undermining area. The distal edges of the wound remained closed and secured with staples (Figure 3).

**Figure 4:**
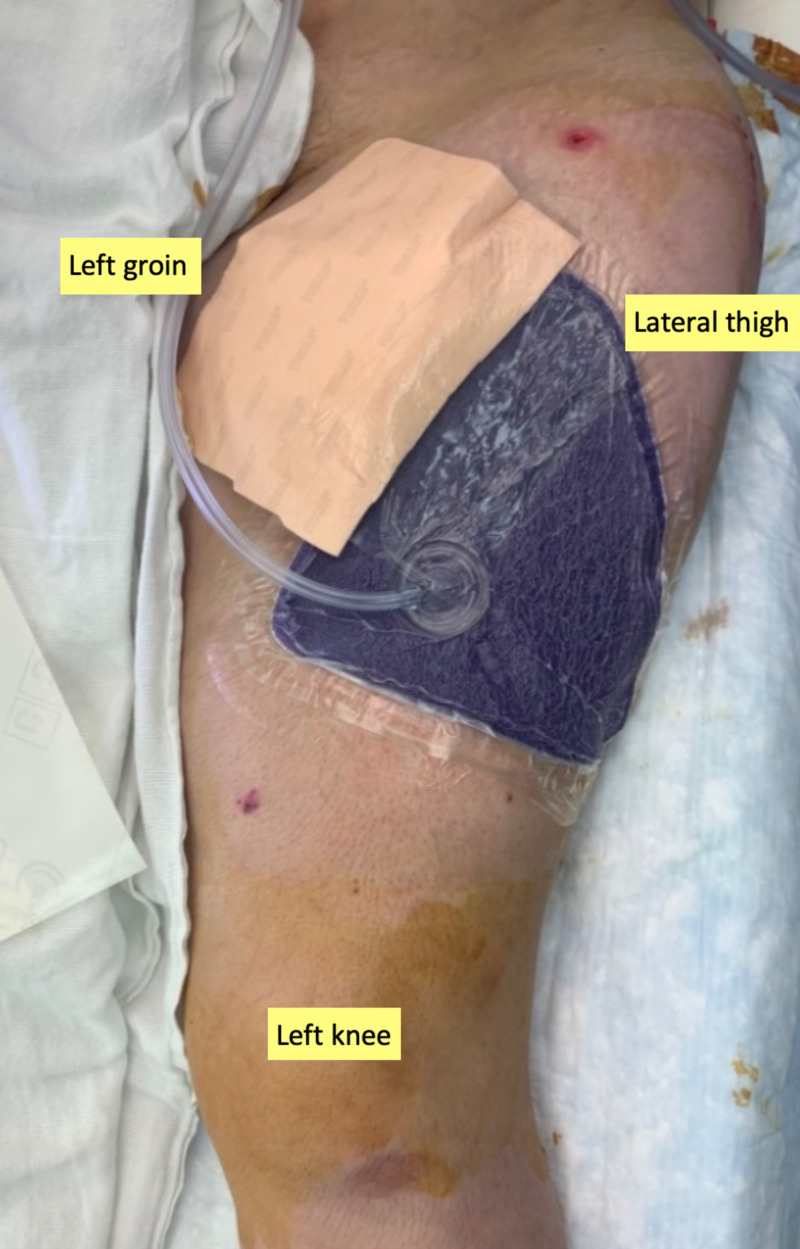
Application of closed incision negative pressure therapy At the close of the final reconstructive surgery, a PREVENA RESTOR BELLA•FORM™ dressing was placed to support the skin graft and re-approximated wound edges.

The exposed distal area of the wound was then covered with a STSG measuring 4 x 3 cm. Several pieces of EpiFix® (MiMedx, Marietta, GA ), a type of tissue matrix allograft, were placed over the skin graft donor site and recipient site, and the donor site was covered with Tielle™ (3M + KCI), a hydropolymer adhesive dressing. A ciNPT dressing (PREVENA™ Therapy; 3M + KCI) set to −125 mm Hg was then placed overlying the re-approximated wound edges and skin graft (Figure 4). The goal was to help with tissue perfusion, protect from external contaminants, strengthen the re-approximated edges, and allow for graft take by bolstering the graft. The patient was discharged home later that day with the PREVENA RESTOR BELLA•FORM™ System (3M + KCI).

**Figure 5:**
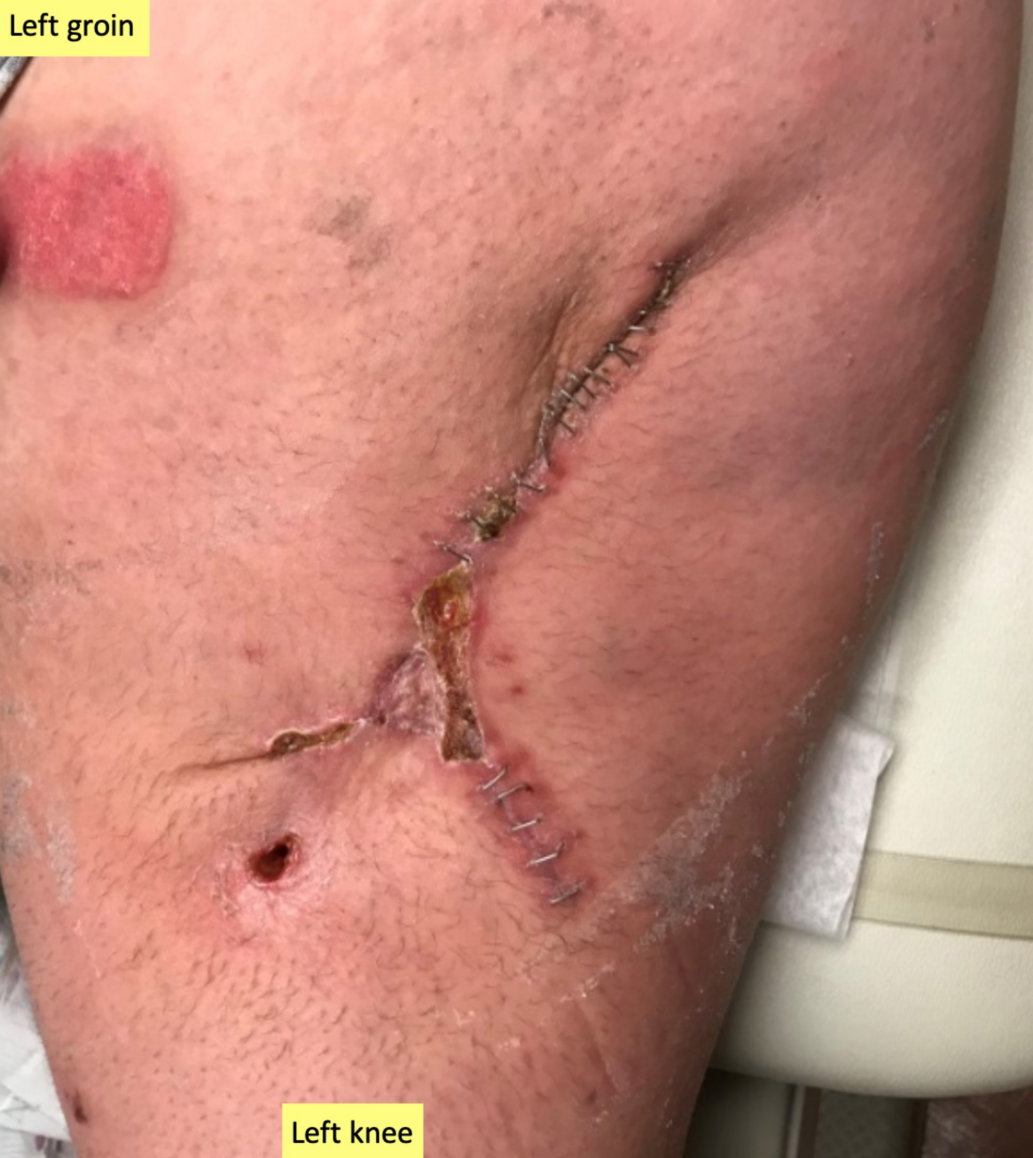
Wound at four weeks post-operatively Wound edges are well-approximated, intact, and strengthened enough for staple removal; skin graft appears viable.

After seven days of ciNPT, the patient was evaluated in the clinic and the ciNPT dressings were removed. On removal of the dressings, the skin graft appeared viable. The wound edges also appeared well-approximated, dry, and intact. Therefore, it was decided to discontinue treatment with the PREVENA RESTOR BELLA•FORM™ System. Non-adherent silicone dressings (ADAPTIC TOUCH™ Non-Adhering Silicone Dressing; 3M + KCI) were placed over the skin graft recipient site, followed by abdominal pads. These were secured in place with an adhesive tape. The patient returned to the clinic once a week for wound evaluation and dressing changes, while also performing dressing changes frequently at home. At four weeks post-operatively, the wound appeared well-approximated with normal scabbing, so staples were removed (Figure 5).

**Figure 6:**
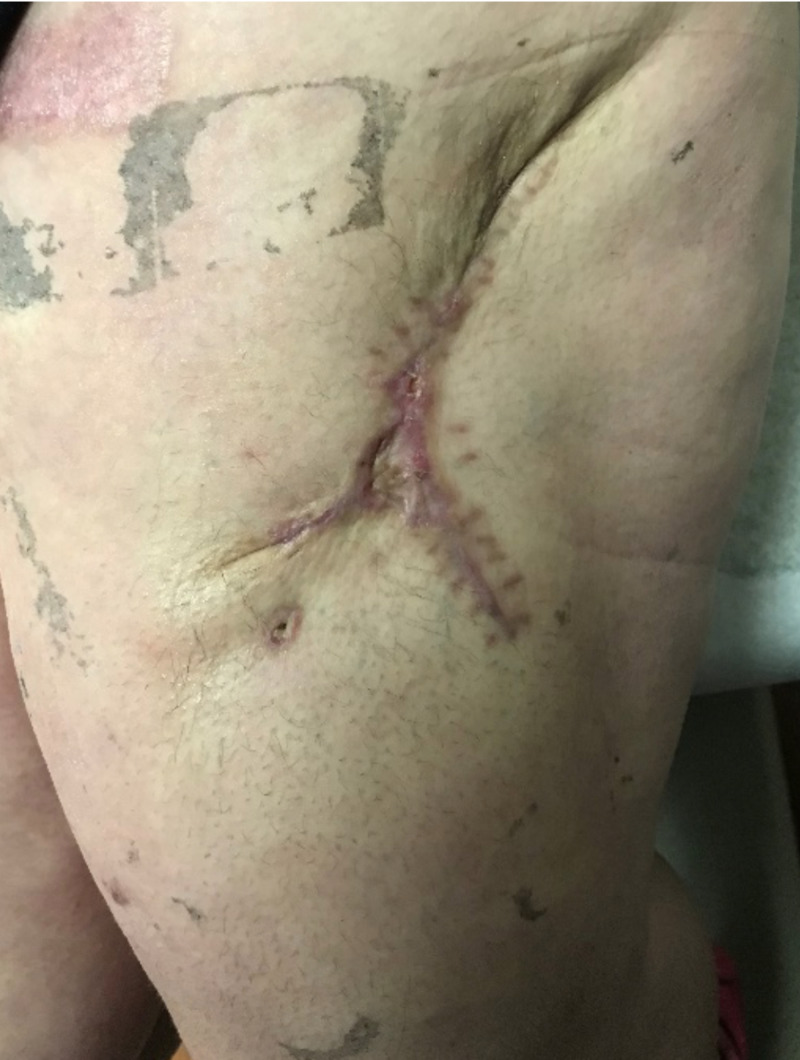
Wound at six weeks post-operatively

At six weeks post-STSG placement and delayed primary closure, the wound remained well-healed with minimal scabbing (Figure 6).

## Discussion

This patient’s complex wound presented many challenges, including a history of necrotizing infection with MSSA, poor tissue perfusion, and resultant soft tissue loss due to her degloving incident. In complicated cases such as this, closure with a soft tissue flap can restore the soft tissue loss and accelerate the process of wound healing [1]. Survival of the repositioned tissue or flap is critical; however, the presence of poor vascularity, soft tissue deficit, or edema can compromise this. In this patient's complex wound, there were progressive signs of vascular failure, including dusky-appearing wound edges and discoloration of the surrounding soft tissue. If the local flap were unable to survive, a free flap reconstruction may be required to achieve successful coverage and closure. This is notoriously a more invasive method of reconstruction, and often avoided if possible.

In this case study, the first priority was to decontaminate the wound, followed by collapsing the extensive devolving injury that was present. The key here is to close all of the dead space in an efficient manner without further loss of function. A clean and decontaminated wound will improve soft tissue adherence and decrease the likelihood of serum formation. Lack of decontamination can lead to deleterious effects such as long-term drainage and sinus formation, thus requiring further surgical intervention to manage. NPWTi-d is a modality known to improve wound healing, even in the presence of infection [4]. In this case, NPWTi-d served to decontaminate the wound as well as collapse all deep potential cavities during the serial stages of reconstruction. During each NPWTi-d change, local flaps were advanced to further collapse the pocket and allow for more of an anatomical reconstruction, thus allowing us to avoid the use of a large skin graft for closure. We followed this with the use of ciNPT to assist with physical protection of the wound, re-approximation of the wound edges, and provide bolstering support to the small skin graft.

We use this modality in such cases because it has been associated with the successful resolution of donor site wounds with other types of flaps [5,6]. According to several studies, the adjunctive use of NPWT improves flap survival, including those that are at a high risk of failure. In a porcine wound model, the use of NPWT at −125 mm Hg was associated with a fourfold increase in laser Doppler-measured blood flow, as well as enhanced flap survival when compared to controls [7]. We understand that according to the manufacturer’s instructions, NPWT dressing changes should occur every two to three days. Although we acknowledge this, we must note that no adverse effects were observed from keeping the dressings in place for seven days.

## Conclusions

In our experience, the combined use of NPWTi-d and ciNPT proved a useful adjunct in managing complex wounds. Although we have only demonstrated their use in this single case, we have shown this in larger series and continue to use this combination. We note that these methods not only improve the overall survival and health of the flap and donor site, but also strengthen the re-approximated wound edges and reduce infection risk.

## References

[1] Chan JK, Harry L, Williams G, Nanchahal J (2012). Soft-tissue reconstruction of open fractures of the lower limb: muscle versus fasciocutaneous flaps. Plast Reconstr Surg.

[2] Zhanjun M, Zonghuan L, Kangquan S (2017). Negative pressure wound therapy: regulating blood flow perfusion and microvessel maturation through microvascular pericytes. Int J Mol Med.

[3] Goldstein JA, Iorio ML, Brown B, Attinger CE (2010). The use of negative pressure wound therapy for random local flaps at the ankle region. J Foot Ankle Surg.

[4] Brinkert D, Ali M, Naud M, Maire N, Trial C, Téot L (2013). Negative pressure wound therapy with saline instillation: 131 patient case series. Int Wound J.

[5] Harris BN, Bewley AF (2016). Minimizing free flap donor-site morbidity. Curr Opin Otolaryngol Head Neck Surg.

[6] Schmedes GW, Banks CA, Malin BT, Srinivas PB, Skoner JM (2012). Massive flap donor sites and the role of negative pressure wound therapy. Otolaryngol Head Neck Surg.

[7] Morykwas MJ, Argenta LC, Shelton-Brown EI, McGuirt W (1997). Vacuum-assisted closure: a new method for wound control and treatment: animal studies and basic foundation. Ann Plast Surg.

